# Ketamine Safety and Use in the Emergency Department for Pain and Agitation/Delirium: A Health System Experience

**DOI:** 10.5811/westjem.2019.10.43067

**Published:** 2020-01-27

**Authors:** Hanjie Mo, Matthew J. Campbell, Baruch S. Fertel, Simon W. Lam, Elizabeth J. Wells, Elizabeth Casserly, Stephen W. Meldon

**Affiliations:** * Cleveland Clinic, Department of Pharmacy, Cleveland, Ohio; † Cleveland Clinic, Emergency Services Institute, Cleveland, Ohio

## Abstract

**Introduction:**

Two protocols were developed to guide the use of subdissociative dose ketamine (SDDK) for analgesia and dissociative sedation ketamine for severe agitation/excited delirium in the emergency department (ED). We sought to evaluate the safety of these protocols implemented in 18 EDs within a large health system.

**Methods:**

We conducted a retrospective chart review to evaluate all adult patients who received intravenous (IV) SDDK for analgesia and intramuscular (IM) dissociative sedation ketamine for severe agitation/excited delirium in 12 hospital-based and six freestanding EDs over a one-year period from the protocol implementation. We developed a standardized data collection form and used it to record patient information regarding ketamine use, concomitant medication use, and any comorbidities that could have impacted the incidence of adverse events.

**Results:**

Approximately 570,000 ED visits occurred during the study period. SDDK was used in 210 ED encounters, while dissociative sedation ketamine for severe agitation/excited delirium was used in 37 ED encounters. SDDK was used in 83% (15/18) of sites while dissociative sedation ketamine was used in 50% (9/18) of sites. Endotracheal intubation, non-rebreather mask, and nasal cannula ≥ four liters per minute were identified in one, five, and three patients, respectively. Neuropsychiatric adverse events were identified in 4% (9/210) of patients who received SDDK.

**Conclusion:**

Patients experienced limited neuropsychiatric adverse events from SDDK. Additionally, dissociative sedation ketamine for severe agitation/excited delirium led to less endotracheal intubation than reported in the prehospital literature. The favorable safety profile of ketamine use in the ED may prompt further increases in usage.

## INTRODUCTION

Ketamine is an *N*-methyl-D-aspartate receptor antagonist that exhibits dissociative sedation and analgesic properties and is commonly used in procedural sedation and induction settings. 1–3 Additionally, ketamine has been explored as novel therapy for analgesia and severe agitation/excited delirium. Multiple studies have described the efficacy and safety of subdissociative-dose ketamine (SDDK) for analgesia in the emergency department (ED), typically with dosing regimens of 0.1–0.3 milligrams per kilogram (mg/kg) administered intravenously (IV). 4–9 Dissociative sedation ketamine, typically defined as 3–5 mg/kg given intramuscularly (IM), 10–12 has been studied for severe agitation/excited delirium in the prehospital setting. 12–14 Ketamine use for this indication has also been studied in a limited number of patients in single-center EDs.^15^,^16^ Adequate sedation is necessary to prevent severe agitation/excited delirium complications such as metabolic abnormalities, cardiac arrest, and death. 11

However, ketamine administration may contribute to serious respiratory, cardiovascular, and neuropsychiatric adverse events.^8^,^10^,^12^ Cole et al. reported intubation rates of 39% for severely agitated patients who received ketamine 5 mg/kg IM vs 4% for those who received haloperidol 10 mg IM in the prehospital setting. 13 These authors also reported intubation rates of 57% in profoundly agitated patients who received ketamine in the prehospital setting. 14 SDDK use may also lead to neuropsychiatric adverse events such as mood changes, dysphoria, confusion, and hallucinations.^4^–^6^, ^16^ To assist emergency medicine (EM) prescribers in safely using ketamine, our emergency services attending physicians and pharmacists developed two ED ketamine protocols for these two indications.

Previous single-center studies have described the benefits and risks of SDDK for analgesia and dissociative sedation ketamine for severe agitation/excited delirium, but the optimal dosing range and administration method of SDDK to minimize adverse effects is unclear.^8^–^9^, ^17^–^18^ Additionally, limited, single- center studies have assessed the safety of IM ketamine for severe agitation/excited delirium in the prehospital setting and ED.^12^–^16^ We designed this multicenter study to provide further insight into the safety of ketamine use in the ED for these two novel indications across a broad spectrum of EDs in a variety of settings.

Our goal was to evaluate the safety of SDDK for analgesia and dissociative sedation ketamine for severe agitation/excited delirium in patients at 18 EDs of a large, integrated health system. The primary objective of this study was to describe the incidence of serious respiratory and cardiovascular adverse events requiring intervention within two hours after ketamine administration. Secondary objectives included describing the incidence of neuropsychiatric adverse events after SDDK administration during the ED encounter; determining the percentage of ketamine orders in the ED for analgesia or severe agitation/excited delirium that were adherent to the approved protocols; and evaluating real-world ketamine use in a large, integrated health system with a diverse group of providers.

### Materials and Methods

This study was a multicenter, retrospective, electronic medical chart review that evaluated the safety of SDDK for analgesia and dissociative sedation ketamine for agitation/excited delirium in ED settings. The authors of this study have no conflict of interest, and our institutional review board approved this study. The study sites included 12 hospital-based EDs and six freestanding EDs with a combined annual census of over half a million ED visits. The hospital-based EDs include a quaternary care, academic medical center, a Level 1 trauma center, and 10 community hospitals, including two Level 2 trauma centers, in both suburban and urban locations. Medical care at the study sites was provided by emergency physicians, medical residents, advanced practice registered nurses, and physician assistants. The health system uses a comprehensive, integrated electronic health record (EHR) (EPIC, Verona, WI) at all hospital-based and freestanding EDs.

**Population Health Research Capsule**

What do we already know about this issue?

The use of subdissociative dose ketamine (SDDK) and dissociative sedation ketamine may lead to serious respiratory, cardiovascular, and neuropsychiatric adverse events.

What was the research question?

To describe the incidence of severe adverse events after ketamine use in the ED and adherence to ketamine dosing protocols.

What was the major finding of the study?

Dissociative sedation ketamine led to less intubation than reported in pre-hospital studies and SDDK led to limited toxicities.

How does this improve population health?

Ketamine used for analgesia or severe agitation/excited delirium leads to limited adverse events and is a viable option when dosed based on our health system’s ketamine protocols.

In 2017 two ketamine protocols were developed to provide guidance for the use of SDDK for acute pain and IM ketamine for dissociative sedation for severe agitation/excited delirium at all EDs across the enterprise. The protocols included indications for therapy as well as recommended dosage regimens and monitoring parameters. See Table 1 for treatment protocol details and treatment indication definitions. The ketamine protocol recommendations were developed by our institution’s emergency physicians, pharmacists, midlevel providers, and nurses based on currently available literature for ketamine use for these two indications and ketamine’s pharmacokinetic and pharmacodynamics profile.

Emergency providers and nursing were educated via email communication and staff meetings regarding the data supporting the new ketamine protocols and the operational changes associated with them. Afterward, the ketamine protocols were implemented on May 9, 2017. SDDK ketamine would be dosed 0.2–0.3 mg/kg, maximum dose 25 mg, as a slow IV push over five minutes with a potential repeated dose in 30 minutes. Ketamine for severe agitation/delirium would be dosed 4 mg/kg IM once, with a maximum dose of 500 mg. Providers were reminded monthly of the new protocol doses and indications through emails, especially with consideration of the opioid crisis and the desire to use alternative, non-opioid analgesics.

Adult patients, at least 18 years old, who received IV SDDK for analgesia and/or IM dissociative sedation ketamine for severe agitation/excited delirium at any study ED from May 9, 2017–May 9, 2018, were included in the study. Exclusion criteria included the following: age <18 years old; administration of ketamine for indications other than analgesia or severe agitation/excited delirium (e.g., rapid sequence intubation, procedural sedation, etc.); or administration of ketamine via route other than IV or IM. Four of the ED sites (one hospital-based ED and three freestanding EDs) were not using the integrated health record until September 29, 2017; therefore, patients were eligible for study inclusion only between September 29, 2017–May 9, 2018, at these four study EDs. An ED agitation protocol order set was built into our EHR that enabled the EM provider to select severe agitation/excited delirium as the indication, which would provide a calculated dose of ketamine 4 mg/kg IM if this drug were selected. A specific order set was not created for ketamine used for analgesia.

We conducted a query of our EHR to identify all patients who received either IV bolus or IM ketamine at a study ED within the study period. A standardized electronic data collection form was developed within Research Electronic Data Capture (REDCap, Nashville, TN), a secure data collection tool. A single investigator, H.M., a clinical pharmacy resident, manually conducted chart review within the EHR to collect data points such as ketamine regimen details; vital sign data; psychiatric comorbidities; concomitant medications (benzodiazepines, antipsychotics, opioids, and antihistamines) administered within one hour before and two hours after ketamine use; predefined ketamine-related adverse events; and additional relevant points for all eligible patients. Adverse event data was identified through review of physician and allied health documentation during the ED visit, as well as review of the medication administration record and respiratory documentation flowsheets within the EHR. Data collected in the respiratory documentation flowsheets included the patient’s respiratory status (eg, endotracheal intubation [ETI], bag valve mask, bilevel positive airway pressure [BiPAP], non-rebreather mask, nasal cannula [NC], or room air), and the timing of respiratory intervention, if applicable. A single investigator collected and reviewed all data to ensure consistency in data interpretation.

The primary outcome was to describe the incidence of severe respiratory and cardiovascular adverse events after ketamine administration. We defined severe respiratory adverse events as use of an advanced airway such as ETI, non-invasive positive pressure ventilation (bag valve mask, BiPAP, continuous positive airway pressure), non-rebreather mask, and/or escalation from baseline oxygen requirements to at least four liters (L) or more per minute via NC within two hours following administration of ketamine dose. Severe cardiovascular adverse events were defined as elevated blood pressure requiring IV antihypertensive medication(s) or new dysrhythmia requiring electrical cardioversion or pharmacological rate and/or rhythm control within two hours following administration of a ketamine dose.

Secondary outcomes included describing the incidence of neuropsychiatric adverse events after SDDK for analgesia administration and determining the percentage of ketamine orders for both indications in the ED that were adherent to the approved protocols. We defined neuropsychiatric adverse events as hallucinations, dysphoria, delusions, and/or any other mood change documented at any time during the ED visit following administration of a ketamine dose. Protocol adherence was defined as administering ketamine without protocol contraindications and using dosing regimens within the approved dose ranges and frequencies. There were no contraindications listed for using ketamine for severe agitation/excited delirium since acute interventions for this emergent condition are time sensitive, and an accurate medical history is often difficult to obtain. We analyzed all data descriptively.

## RESULTS

During the study period, we identified 762 ED encounters with ketamine administration; 515 did not meet study inclusion criteria. A total of 247 ED encounters (210 SDDK and 37 dissociative sedation ketamine) were included in the study. There were 170 unique patient encounters as 13 patients within the SDDK group had repeated ED visits. These 13 patients accounted for 53/210 (25.2%) of all SDDK ED encounters. All patient characteristics reported were calculated based on unique patient encounters. The median age was 43 years of age (interquartile range [IQR]: 30–54) in the SDDK group and 39 (IQR: 31–48) in the dissociative sedation group. Median baseline blood pressure was 130/81 millimeters of mercury (mmHg) (IQR: 118–149.5/71–90.5 mmHg) prior to ketamine administration in the SDDK group. The most frequently used concomitant medications for the SDDK group were opioids (30%), while benzodiazepines (54.1%) were more commonly used in the dissociative sedation ketamine group. Table 2 summarizes patient demographics for each ketamine group.

In the SDDK group, the median ketamine dose was 20 mg (IQR: 16.1–25) IV with a median weight-based dose of 0.26 mg/kg (IQR: 0.2–0.3) IV. In the severe agitation/excited delirium group, the median ketamine dose was 242.4 mg (IQR: 124.7–319.4) IM with a median weight-based dose of 3.2 mg/kg (IQR: 1.89–4.0) IM. Fifteen out of 18 (83%) ED sites used SDDK for analgesia while 9/18 (50%) used dissociative sedation ketamine for severe agitation/delirium (Table 3). The distribution of ketamine use by type of ED is also described in Table 3.

For the primary outcome, serious respiratory adverse events were identified in 1% (2/210) of patients in the SDDK group and 16.2% (6/37) of patients in the dissociative sedation group. Additionally, serious cardiovascular adverse events were identified in 0.5% (1/210) of patients in the SDDK group. Examples from the SDDK group included three different patients who either received NC oxygen ≥ 4 L/minute (min), required a non-rebreather mask, or were given IV antihypertensive therapy for elevated blood pressures. Examples from the dissociative sedation group included one patient who received ETI, three patients who received a non-rebreather mask, and two patients who received NC oxygen ≥ 4 L/min. The patient who received ETI initially came in with seizures and had no history of substance abuse or psychosis documented.

In the SDDK group 4.3% (9/210) of patients experienced neuropsychiatric adverse events. Patients described these neuropsychiatric adverse events as feeling “out of it,” “uncontrolled,” confused, and anxious. One patient experienced hallucinations. The same patient who experienced the serious cardiovascular adverse event also experienced an emergence reaction and neuropsychiatric adverse event. No neuropsychiatric adverse events required intervention. Adverse events are summarized in Tables 4–6.

For the secondary outcome of protocol adherence, 80% of patients (167/210) in the SDDK group and 32% (12/37) in the dissociative sedation group met adherence criteria (administering SDDK without protocol contraindications or ketamine for either indications within the approved dose ranges and frequencies). Eight patients who received SDDK had a systolic blood pressure greater than 180 and one patient presented with head trauma, both of which are protocol contraindications for SDDK. A total of 11.4% of patients (24/210) in the SDDK group and 54.1% (20/37) in the dissociative sedation ketamine group received doses below the recommended range. The recommended dose is 0.2–0.3 mg/kg IV for SDDK, maximum dose 25 mg, and can be repeated once in 30 minutes, and 4 mg/kg IM once for severe agitation/excited delirium. Additionally, 3.8% of patients (8/210) in the SDDK group received ketamine above 0.3 mg/kg IV or higher than the maximum recommended single dose of 25 mg IV. Five of 37 patients (13.2%) received dissociative sedation ketamine doses greater than 4 mg/kg IM. A summary of ketamine protocol adherence is described in Table 7.

## LIMITATIONS

This study was retrospective and relied on accurate documentation of adverse events. Variable documentation may have impacted the results identified. However, this limitation was minimized by using objective outcome measures such as specific respiratory and medication interventions to define adverse events. Neuropsychiatric adverse events may also have been under-reported considering that ED nursing, respiratory therapy, and/or physician documentation was the primary source for identification. The descriptiveness of these side effects reported were also limited by chart documentation. However, none of the identified neuropsychiatric adverse events required intervention, suggesting that the adverse effects observed were mild and self-limiting. Additional limitations include the data review by a single abstractor, who potentially may have not captured all adverse events. Furthermore, there was no control group to calculate a confidence interval and determine whether these adverse events identified were statistically significant. Last, data was not available to further characterize patients who met exclusion criteria.

## DISCUSSION

In this multicenter pragmatic study, the use of SDDK for analgesia and dissociative sedation dosing for severe agitation/excited delirium resulted in a lower incidence of serious respiratory adverse events than previously reported. In patients receiving dissociative sedation ketamine for severe agitation/excited delirium, 16.2% of patients experienced serious respiratory adverse events. However, only one of these patients required ETI yielding an intubation rate of only 2.7% within this treatment group. In contrast, Cole et al.’s single-center studies reported that intubation rates were 39% in severely agitated patients and 57% in profoundly agitated patients who received ketamine 5 mg/kg IM in the prehospital setting. 13–14 These studies raised concern about the safety of ketamine use. The differences in intubation rates between our study and Cole et al.’s studies may be attributed to a number of factors. Ketamine administration in the prehospital setting for severe agitation/excited delirium results in the arrival of a fully dissociated patient at the time of ED presentation, which may contribute to a higher probability of a respiratory intervention provided by the receiving emergency physician.

In our study, patients were evaluated by an emergency physician prior to the institution of dissociative sedation, which may have allowed for more appropriate selection of patients and closer monitoring of patients’ respiratory status following administration of ketamine. The patients in our study also received a lower median dose of 3.2 mg/kg IM (IQR: 1.9–4.0 mg/kg), compared to an average of 5 mg/kg in the Cole et al. studies. The lower ketamine doses also may have led to a decreased incidence of respiratory depression requiring ETI. Furthermore, the use of concomitant medications with sedative potential in the prehospital setting may have also led to an increased number of respiratory adverse events. Cole et al. reported that patients in their study had positive urine drug screen results for opioids (21%), diphenhydramine (12%), antipsychotics (15%), and benzodiazepines (18%). 13 The amount, frequency, and timing of these administrations is unknown.

Although our study did not evaluate the use of prehospital medications, only 5.4% of our patients received opioids one hour before or two hours after ketamine use in the ED. Of note, controversy does exist as to whether intubation itself is considered an adverse event given that these patients were severely agitated and at high risk of harm to self or others where the only alternative was to intubate and sedate to ensure patient safety. Finally, the increased publicity around ketamine usage in the ED literature may have led to more familiarity with its pharmaceutical properties and concomitant risks. Our protocol required that once sedation occurred, a full complement of monitoring be deployed including cardiac monitoring, pulse oximetry, and presence of nursing at bedside, similarly to procedural sedation practices.

SDDK use led to few serious respiratory adverse events requiring intervention. One patient required NC ≥ 4 L/min and a second patient required the use of a non-rebreather mask; none of the patients required ETI. Both of these patients received just one dose of ketamine and met all protocol adherence criteria. However, both patients also received one dose of fentanyl IV 50 micrograms and morphine IV 4 mg, respectively. Additionally, one of these patients had a history of drug and/or alcohol abuse, which may have impacted the occurrence of adverse events. Although the use of opioids and having a history of drug and/or alcohol abuse may have contributed to the need for respiratory intervention, these findings suggest that close patient monitoring of respiratory status with SDDK may be warranted.

One patient in the SDDK group (0.5%) also experienced elevated blood pressures following ketamine administration, which required IV antihypertensive therapy despite the absence of any protocol contraindications. This patient’s blood pressure went from a baseline of 161/86 mmHg to 205/164 mmHg after ketamine administration. The potential for ketamine to contribute to elevated blood pressure and heart rate has been well established. Review of vital signs prior to dose administration to determine the absence of hemodynamic instability and monitoring vital signs after dose administration is indicated.

SDDK use also led to limited and non-severe neuropsychiatric adverse events in 4.3% of patients. However, Motov et al. reported higher rates of mood changes (13%) and disorientation (29%) in patients who received 0.3 mg/kg (mean 21.8 mg, standard deviation 4.9 mg) of ketamine IV push over 3–5 minutes.^8^ In contrast, Sin et al. reported neuropsychiatric adverse advents in 3% of patients who received ketamine IV 0.3 mg/kg IV piggyback over 15 minutes.^16^ Neither study included patients with a past medical history of psychiatric illness or substance abuse or described concomitant medications used outside of the protocol.^8^,^16^ Concomitant medications and comorbidities may lead to an increase in adverse events.

Due to the retrospective design of our study, the lower incidence of neuropsychiatric adverse events reported may have been attributed to incomplete documentation of adverse events in the EHR. The lower rates of neuropsychiatric adverse events in our study may also have been due to our ketamine single-dose cap of 25 mg and the requirement of a slow IV push administration over at least five minutes. Our multicenter study results support that SDDK use led to minor neuropsychiatric adverse events that did not require intervention, which aligns with previously published, single-center studies.^5^,^8^

Despite frequent reminders, the protocols were not used at all of the ED sites in our enterprise. This probably reflects the reality of knowledge translation and willingness of more experienced physicians to try new medications with which they are less familiar or their comfort with more conventional therapy for analgesia such as opioids or benzodiazepines and haloperidol for agitation. While ketamine usage has been increasingly touted in Free Open Access Medication Education (FOAMed) and other social media, the vocal users may be in more academic settings or accustomed to trying novel therapies. The strength of our study lies in its real-world experience across a diverse group of providers and ED sites. This study should help alleviate some concerns of those providers that the therapy is safe and effective even in small EDs.

The protocol adherence for SDDK was 80%, while dissociative sedation ketamine was 32%. The decreased protocol adherence was attributed primarily to patients receiving ketamine below the recommended doses. This can paradoxically be harmful as partially dissociated patients have more neuropsychiatric effects, which may increase agitation. This is perhaps due to the ED prescribers not being as comfortable with ordering higher doses of ketamine. Additionally, it is difficult to collect an accurate weight for severely agitated patients who present to the ED; thus, empiric dosing may have been based on estimated weight for a number of patients, potentially contributing to dosing variance beyond the protocol-recommended dosing range.

Several patients also received ketamine doses above the recommended range. This may not portend harm as once dissociation is achieved, there is no further depth to sedation with increased ketamine administration. Additionally, protocol variance may have also been impacted by the specific ED site and emergency physician. Three out of the 18 EDs accounted for 70% of IM ketamine use for severe agitation/excited delirium. This protocol required attending physician administration of the drug, which may have limited its use.

## CONCLUSION

Dissociative sedation ketamine dosed at 4 mg/kg IM for severe agitation can result in serious respiratory adverse events. However in our experience, this occurred less frequently than previously reported in single-center studies. When used at subdissociative-doses for analgesia at 0.2–0.3 mg/kg and administered as slow IV push over five minutes, ketamine is associated with minor and self-limited neuropsychiatric adverse events that resolve without further intervention. In summary, the overall favorable safety profile of ketamine use as described in our experience in a number of diverse ED settings supports a more widespread use of SDDK and dissociative dosing for acute agitation. Further research is needed to address barriers that prevent more extensive usage of ketamine by ED providers.

## Figures and Tables

**Table 1:** Ketamine treatment protocols.

	Subdissociative-dose ketamine (SDDK) for analgesia	Ketamine for severe agitation/excited delirium
Indications	First-line analgesic therapy for management of severe pain in the following scenarios: Acute pain secondary to traumatic injury Acute pain in patients with documented allergy/intolerance to parenteral opioid therapy Chronic pain in patients who are not candidates for opioid or NSAID therapy Adjunct analgesic therapy for the management of severe pain in ED patients who failed to achieve therapeutic response with parenteral opioid therapy	First-line pharmacologic monotherapy for adult patients with severe agitation, excited delirium, and violent/self-destructive behavior who meet the following criteria: Patient poses an immediate threat to patient and healthcare provider safety (RASS score of +4) Failure and/or futility of alternative non-pharmacologic de-escalation strategies Absence of IV access Not a candidate for intramuscular antipsychotics and/or benzodiazepines due to unacceptably protracted onset of action
Contraindications	Unstable vital signs Systolic blood pressure > 180 mmHg Heart rate > 150 beats per minute Respiratory rate < 10 or > 30 Suspected acute coronary syndrome Acute decompensated heart failure Unstable dysrhythmia Acute head or ocular trauma Suspected elevated intracranial pressure History of schizophrenia or other psychosis Active psychosis	None
Dosing regimen and administration	0.2 – 0.3 mg/kg (up to a max dose of 25 mg) Administered as slow IV push over 5 minutes Dose may be repeated once in 30 minutes	4 mg/kg IM up to max single dose of 500 mg Dosing weight can be estimated if actual weight unavailable Immediate availability of advanced airway equipment
Patient monitoring	Vital signs (including pain assessment) at baseline, 15 minutes, and 30 minutes after each dose followed by routine nursing care per department protocol Continuous pulse oximetry for at least 30 minutes after dose administration Telemetry for at least 30 minutes after dose administration Immediate availability of ED attending physician for at least 30 minutes	Continuous direct patient observation for minimum of 15 minutes Continuous pulse oximetry, cardiac monitor, and end-tidal CO2 monitoring (if available) Removal of physical restraints Supine patient positioning with elevation of head of bed at 30°

*ED*, emergency department; *NSAID*, Nonsteroidal anti-inflammatory drugs; *RASS*, Richmond Agitation-Sedation Scale; *IV*, intravenous; *mmHg*, millimeters of mercury; *mg/kg*, milligrams per kilogram; *CO_2_*, carbon dioxide.

**Table 2:** Baseline patient demographics.

Patient characteristics	SDDK IV for analgesia (n=210)	Ketamine IM for agitation/delirium (n=37)
No. of ED encounters	210	37
Age, median (IQR), years	43 (IQR: 30–54)	39 (IQR: 31–48)
Sex, male (%)	39	70
Weight, median (IQR), kg	79.9 (65.8–90.7)	77.1 (IQR: 68–99.8)
History of psychosis (%)	11.9	40.5
History of illicit drug use or alcohol abuse (%)	22.4	59.5
Systolic blood pressure, median (IQR), mmHg*	130 (118–149.5)	N/A
Diastolic blood pressure, median (IQR), mmHg*	81 (71–90.5)	N/A
Pulse rate, median (IQR), beats/min*	83 (IQR: 70–98)	N/A
O_2_ saturation, median (IQR), %*	98 (IQR: 97–100)	N/A
Concomitant medications† (%)		
Antihistamines	10.0	24.3
Antipsychotics	3.3	35.1
Benzodiazepines	12.4	54.1
Opioids	30.0	5.4

* Baseline vitals prior to ketamine administration

† One hour before or two hours after ketamine administration

*SDDK*, subdissociative-dose ketamine; *IV*, intravenous; *IM*, intramuscular; *ED*, emergency department; *IQR*, interquartile range; *kg*, kilogram; *mmHG*, millimeters of mercury; *O_2_*, oxygen.

**Table 3:** Ketamine utilization.

Ketamine	SDDK IV for analgesia	Ketamine IM for agitation/delirium
Dose in mg, median (IQR)	20 (IQR: 16.1–25)	242.4 (IQR 124.7–319.4)
Dose in mg/kg, median (IQR)	0.26 (0.2–0.3)	3.2 (1.89–4)
Unique ED encounters	170	37
No. patients with repeated ED encounters	13, accounting for 40 ED encounters	0
No. ED locations using ketamine (%)	15 out of 18 (83)	9 out of 18 (50)
ED Type		
Hospital-based ketamine patients	196	37
Freestanding ketamine patients	14	0

*SDDK*, subdissociative-dose ketamine; *IV*, intravenous; *IM*, intramuscular; *ED*, emergency department; *IQR*, interquartile range; *mg*, milligram; *kg*, kilogram; *no.*, number.

**Table 4:** Adverse events.

	SDDK IV for analgesia (n=210)	Ketamine IM for agitation/ delirium (n=37)
Respiratory	2 (1.0%)	6 (16.2%)
Endotracheal intubation	0	1† (2.7%)
Bag valve mask	0	0
BiPAP	0	0
Non-rebreather mask	1 (0.5%)	4† (10.8%)
Nasal cannula O_2_ ≥ 4 L/min	1 (0.5%)	2 (5.4%)
Cardiovascular	1* (0.5%)	0
Neuropsychiatric	9* (4.3%)	0

† Indicates one patient required both non-rebreather mask use and endotracheal intubation

* Indicates one patient experienced a cardiovascular and neuropsychiatric adverse event

*SDDK*, subdissociative-dose ketamine; *IV*, intravenous; *IM*, intramuscular; *BiPAP*, bilevel positive airway pressure.

**Table 5:** Respiratory and cardiovascular adverse event patient cases: dissociative sedation ketamine.

	Patient 1*	Patient 2	Patient 3	Patient 4	Patient 5	Patient 6
Age, years	42	34	54	54	30	31
Gender	Male	Male	Female	Female	Male	Male
Weight, kg	79.4	86.2	55.8	68	162	113.4
History of psychosis	No	No	Yes	Yes	No	Yes
Illicit drug/ alcohol abuse	No	Yes	Yes	Yes	Yes	No
Number of ketamine doses during ED visit	3	1	1	1	1	2
Ketamine dose, mg	238.2 IV, 158.8 IV, and 317.6 IM	300 IM	223.2 IM	300 IM	150 IM	113.4 IM x2
Ketamine dose, mg/kg	2 IV, 3 IV, and 4 IM	3.48 IM	4 IM	4.4 IM	0.92 IM	1 IM x2
Baseline supplemental oxygen	No	No	No	No	No	No
Concomitant medications 1 hour before or 2 hours after ketamine use	Lorazepam IV 1 mg x1 and 2mg x2 Haloperidol IM 5 mg x1	None	Lorazepam IM 2 mg x1 Diphenhydramine IV 50 mg x1	Lorazepam IV 1 mg x2	None	Lorazepam IM 2 mg x1
AE identified	Non-rebreather mask and endotracheal intubation	Non-rebreather mask	Non-rebreather mask	Non-rebreather mask	NC O_2_ ≥ 4 L/min	NC O_2_ ≥ 4 L/min
Time (min) between AE and ketamine use	4 min after first ketamine IV dose and 120 min after IM ketamine dose	12	104	44	41	42 min after second ketamine dose

* Required endotracheal intubation

*Kg*, kilogram; *ED*, emergency department; *mg*, milligram; *IV*, intravenous; *IM*, intramuscular; *AE*, adverse event; *NC*, nasal cannula; *O_2_*, oxygen; *L*, liters; *min*, minute.

**Table 6:** Respiratory and cardiovascular AE patient cases: subdissociative ketamine.

	Patient 1	Patient 2	Patient 3
Age, years	70	28	66
Gender	Male	Male	Female
Weight, kg	69.4	149.7	53.1
Psychosis	No	No	No
Illicit drug/ alcohol abuse	No	Yes	No
Number of ketamine doses	1	1	1
Ketamine dose, mg	20.82 IV	25 IV	15 IV
Ketamine dose, mg/kg	0.3 IV	0.17 IV	0.28
Baseline supplemental oxygen	No	No	No
Concomitant medications 1 hour before or 2 hours after ketamine use	Fentanyl IV 50 mcg x1	Morphine IV 4 mg x1	Morphine IV 5 mg x1
AE identified	Non-rebreather mask	NC O_2_ ≥ 4 L/min	Elevated BP receiving IV labetalol 10 mg x1 (BP increased to 205/164 from 161/86 mmHg)
Time (min) between AE and ketamine use	32	9	76

*Kg*, kilogram; *mg*, milligram; *IV*, intravenous; *mcg*, microgram; *AE*, adverse effect; *NC*, nasal cannula; *L*, liter; *min*, minute; *BP*, blood pressure.

**Table 7:** Ketamine protocol adherence.

Proportion of ketamine regimens adherent to health-system protocols	SDDK IV for analgesia	Ketamine IM for agitation/ delirium
Without protocol contraindications	201/210 (96%) Received ketamine when SBP>180 mmHg: 8 Received ketamine presenting with head trauma: 1	Not applicable
Dosing regimens within the approved dose ranges and frequencies based on our institution’s ketamine protocols (refer to Table 1)	175/210 (83%) Dosed below range: 24 Dosed above range: 8 Received > 2 doses in same ED visit: 2 Received ketamine <30 min apart: 1	12/37 (32%) Dosed below range: 20 Dosed above range: 5
Total adherence	167/210* (80%)	12/37 (32%)

* One patient received ketamine with an SBP > 180 mmHg and above the recommended dose

*SDDK*, subdissociative-dose ketamine; *IV*, intravenous; *IM*, intramuscular; *SBP*, systolic blood pressure; *mmHG*, milligrams of mercury; *ED*, emergency department; *min*, minute.
